# Aberrant amino acid signaling promotes growth and metastasis of hepatocellular carcinomas through Rab1A-dependent activation of mTORC1 by Rab1A

**DOI:** 10.18632/oncotarget.5175

**Published:** 2015-08-13

**Authors:** Bi-Hong Xu, Xiao-Xing Li, Yang Yang, Mei-Yin Zhang, Hui-Lan Rao, Hui-Yun Wang, X.F. Steven Zheng

**Affiliations:** ^1^ State Key Laboratory of Oncology in South China, Collaborative Innovation Center for Cancer Medicine, Sun Yat-sen University Cancer Center, Guangzhou, China; ^2^ Department of Pathology, Sun Yat-Sen University Cancer Center, Guangzhou, China; ^3^ Rutgers Cancer Institute of New Jersey and Department of Pharmacology, Robert Wood Johnson Medical School, Rutgers, The State University of New Jersey, New Brunswick, NJ, USA

**Keywords:** signal transduction, Rab1A, mTORC1, cancer, rapamycin

## Abstract

mTORC1 is a master regulator of cell growth and proliferation, and an established anticancer drug target. Aberrant mTORC1 signaling is common in hepatocellular carcinoma (HCC), but the underlying mechanism remains elusive. Rab1A is a newly identified mTORC1 activator that mediates an alternative amino acid (AA) signaling branch to Rag GTPases. Because liver is a physiological hub for nutrient sensing and metabolic homeostasis, we investigated the possible role of Rab1A in HCC. We found that Rab1A is frequently overexpressed in HCC, which enhances hyperactive AA-mTORC1 signaling, promoting malignant growth and metastasis of HCC *in vitro* and *in vivo*. Moreover, aberrant Rab1A expression is closely associated with poor prognosis. Strikingly, aberrant Rab1A overexpression leads to increased rapamycin sensitivity, indicating that inappropriate activation of AA signaling is a cancer-driving event in HCC. Our findings further suggest that Rab1A is a valuable biomarker for prognosis and personalized mTORC1-targeted therapy in liver cancer.

## INTRODUCTION

HCC is the predominant form of liver cancer. It is one of the most common malignancies in the world and the third leading cause of cancer-related death [1, 2]. Although HCC is most prevalent in East Asia, its incidence has seen significant increase in North America and Western Europe in recent years [3]. Due to lack of obvious symptoms and reliable early detection methods, majority of the patients are diagnosed at an advanced disease stage when there are few curative options [4]. The pathogenesis of HCC is thought to be due to a stepwise accumulation of mutations, genomic aberrations and epigenetic changes, resulting in alterations in the function of genes critical for growth, proliferation, migration, and invasion [5]. Because of the high mortality rate, there is a pressing need to identify key molecular events underlying the development of this disease in order to improve diagnosis and therapy.

mTOR is a PI3K-related kinase conserved throughout the eukaryotic kingdom. It forms two distinct complexes named mTORC1 and mTORC2, which are composed of the mTOR kinase subunit and accessory proteins [6]. mTORC1, a master growth regulator and a nutrient sensor, regulates growth-related processes such as translation and ribosome biogenesis. mTORC2, on the other hand, phosphorylates several AGC kinases including AKT, to promote survival. Due to the central role of mTORC1 in growth, aberrant mTORC1 signaling has been linked to human diseases particularly cancer [7]. Because many tumors are addictive to activated mTOR signaling, mTOR is a desirable target for molecular therapy. Temsirolimus and everolimus, semisynthetic analogs of the highly specific mTORC1 inhibitor rapamycin, are FDA-approved drugs for advanced mammary and renal carcinomas [8]. In addition, mTOR kinase inhibitors are currently in many human cancer clinical trials [8].

mTOR activation promotes rapid cell growth and proliferation [9, 10]. Recent studies indicate that mTOR signaling is aberrantly activated in HCC [11, 12]. Rapamycin (sirolimus) is clinically used as an immunosuppressant for liver transplantation after HCC surgery [13]. Compared with FK506 (tacrolimus), rapamycin-treated patients have significantly higher recurrence-free survival [14–16]. It has been speculated that rapamycin has the benefit of inhibiting graft rejection while simultaneously preventing tumor recurrence. A number of mTORC1-targeted HCC clinical trials have been carried out (clinicaltrials.gov) [17]. However, mTOR inhibitor as a single agent or in combination therapy has achieved limited success to date [17]. Therefore, it is of urgent need to investigate the mechanism of mTOR activation and identify surrogate biomarkers.

mTORC1 is known to be downstream of phosphatidylinositide 3-OH kinase (PI3K). However, recent evidence shows that oncogenic PI3K is often insufficient to promote mTORC1 activity in colorectal and breast cancer cells [18, 19], suggesting that other events such as amino acid signaling are involved. We have previously conducted a genomic rapamycin screen for mTORC1 signaling pathway components in yeast [20], and identified Rab1A, a small GTPase previously known for its role in ER-to-Golgi trafficking, as a critical mediator of AA signaling to activate mTORC1 [21]. In the present study, we investigated Rab1A expression in HCC, and found that it is frequently overexpressed in human primary HCCs, which is correlated with poor prognosis. We further studied the role of Rab1A in mTORC1 signaling and HCC oncogenesis. Our results demonstrate that Rab1A is a new oncogenic protein with potentially significant prognostic and therapeutic values.

## RESULTS

### Rab1A is overexpressed in HCC cells, which is due to gene amplification

**Figure 1:**
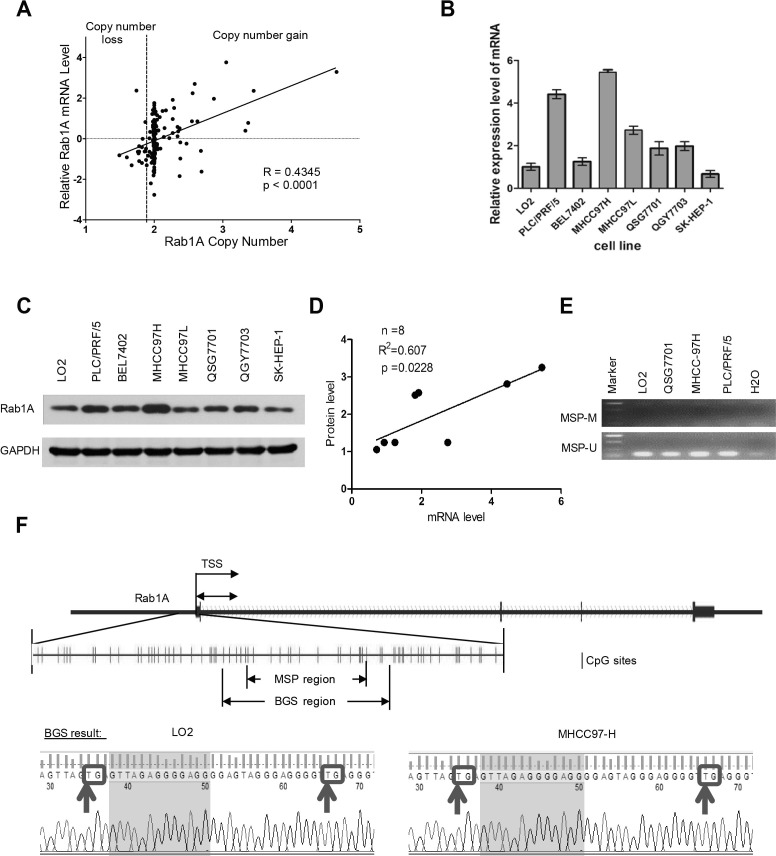
Rab1A is overexpressed in HCC due to copy number increase **A.** Correlation plot of the Rab1A copy number and mRNA level in 187 HCC samples in the TCGA cancer genome database. **B.** Rab1A mRNA expression in a panel of immortalized liver and HCC cell lines as determined by RT-qPCR. **C.** Rab1A protein expression in the same panel of immortalized liver and HCC cell lines as determined by immunoblot analysis. GAPDH served as a loading control. **D.** Correlation plot of Rab1A protein and mRNA expression in the above panel of cell lines. **E.** MSP results for HCC (MHCC97H and PLC/PRF/5) and immortalized liver (LO2 and QSG-7701) cell lines. M, methylated products of MSP; U, unmethylated products of MSP. **F.** Upper panel shows the CpG island used for designing primers to detect methylation status of Rab1A locus. Lower panel shows the atlas of PCR fragments for BGS. Arrows indicate potential methylated sites in the LO2 and MHCC97H cell lines. TSS, transcription start site.

Genome data from two previous studies deposited in The Cancer Genome Atlas (TCGA) suggest that Rab1A gene is amplified in many HCC cases (http://cancergenome.nih.gov) [22, 23]. We therefore examined the relationship between the Rab1A copy number and mRNA expression in 187 HCC samples available in the Cancer Genome Atlas (TCGA) cancer genomic database (http://cancergenome.nih.gov/). Linear regression analysis shows a strong correlation between genomic DNA copy number and mRNA level of Rab1A (Figure 1A). To further understand the molecular basis underlying aberrant Rab1A expression, we examined mRNA and protein expression of Rab1A in two immortalized liver (LO2 and QSG-7701) and six HCC cell lines. Rab1A protein and mRNA levels are both generally higher in HCC cell lines compared to immortalized liver cell lines (Figure 1B and 1C). Rab1A protein and mRNA levels are correlated, suggesting that increased Rab1A is due to change in gene expression (Figure 1D). These results indicate that HCC cell lines resemble those seen with human tumors, and are useful *in vitro* models for studying aberrant Rab1A expression. Epigenetic alteration is another major cause of aberrant gene expression in cancer. However, upon examination of CpG island methylation of Rab1A promoter in two HCC cell lines (MHCC97H and PLC/PRF/5) with high Rab1A expression, and in two immortalized liver cell lines (LO2 and QSG-7701) with low Rab1A expression, we did not find significant DNA methylation in any of the HCC cell lines (Figure 1E and 1F), suggesting that methylation does not contributes significantly to aberrant Rab1A expression.

### Rab1A promotes oncogenic growth of HCC

**Figure 2:**
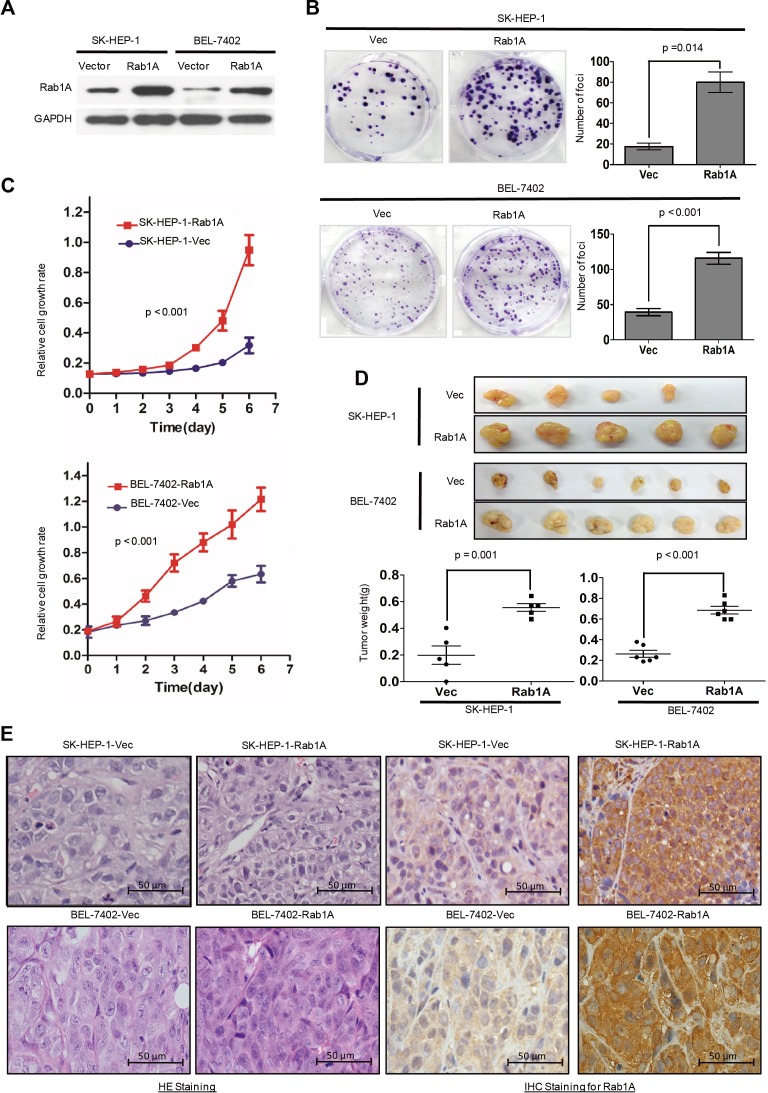
Rab1A overexpression promotes oncogenic growth of HCC *in vitro* and *in vivo* **A.** Ectopic Rab1A expression in SK-HEP-1 and BEL7402 cells. Shown is immunoblot analysis of Rab1A expression in ectopic Rab1A-expressing or control vector cells. *GAPDH* served as a loading control. **B.** Rab1A overexpression promotes colony formation in HCC cells. SK-HEP-1 and BEL7402 cells overexpressing Rab1A or carrying a control vector were assayed for their ability to form colonies. Results are expressed as mean ± SD of three independent experiments. **C.** Rab1A overexpression promotes the growth of HCC cells. SK-HEP-1 and BEL7402 cells overexpressing Rab1A or carrying a control vector were analyzed for growth using the CCK-8 assay. Results are expressed as the mean ± SD of three independent experiments. **D.** Rab1A overexpression promotes HCC tumor growth in xenograft nude mice. Upper panels show images of xenograft tumors at the end of study in nude mice that received a subcutaneous injection of SK-HEP-1 and BEL7402 cells overexpressing Rab1A or carrying a control vector. Lower panels show weights of individual tumors in the two groups. **E.** H&E and IHC staining for Rab1A in Rab1A-overexpressing and control tumors derived by SK-HEP-1 and BEL-7402 (magnification 200 ×).

Rab1A is important for AA to activate mTORC1, a central regulator of cell growth. We investigated the consequence of Rab1A overexpression by stably expressing Rab1A in SK-HEP-1 and BEL-7402 (Figure 2A), two HCC cell lines with similarly low endogenous Rab1A expression to immortalized liver cell lines LO2 and QSG-7701. Strikingly, moderate ectopic Rab1A expression (∼2-3-fold of endogenous Rab1A) is sufficient to robustly increase the rate of colony formation and cell growth compared with control cells (Figure 2B and 2C). In xenograft mouse models established by injecting SK-HEP-1-Rab1A or BEL-7402-Rab1A cells into the right dorsal flanks and subcutaneously vector control cells into the left dorsal flank of the same animals, tumor burden with Rab1A overexpression is significantly larger than control tumors (Figure 2D). IHC staining reveals that Rab1A-overexpressing tumors have higher cell density, mitotic index and nuclear variability, indicating that these tumors are more malignant than control tumors (Figure 2E). Our results demonstrate that Rab1A overexpression promotes oncogenic growth and proliferation *in vitro* and *in vivo*.

### Rab1A promotes HCC cell migration, invasiveness and metastasis

**Figure 3:**
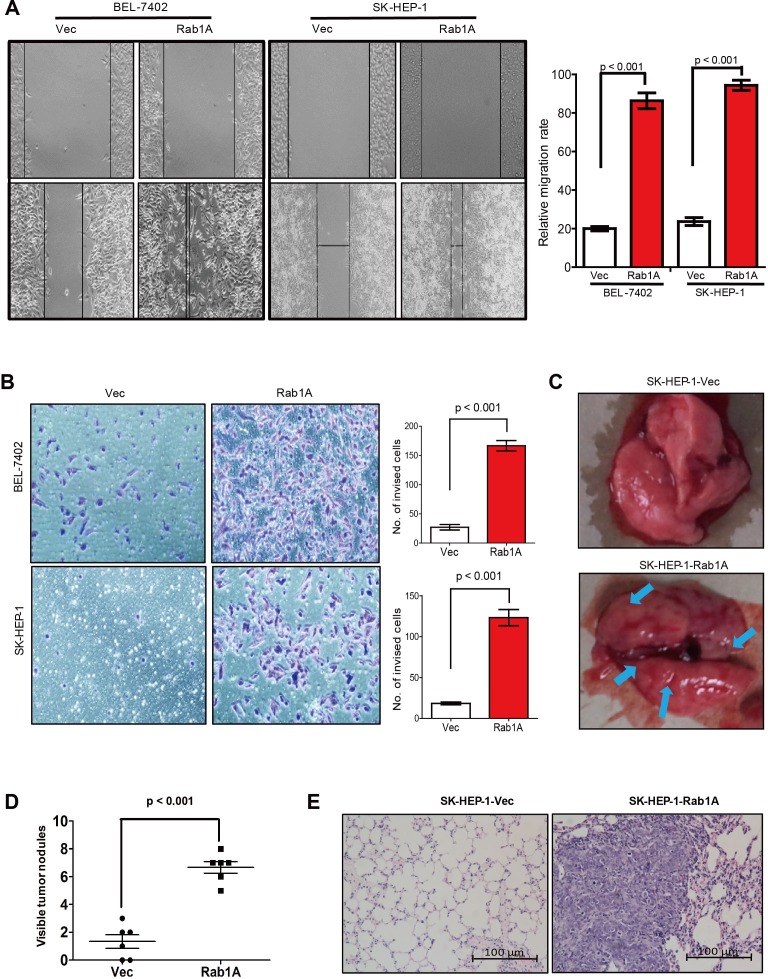
Rab1A overexpression promotes HCC cell migration, invasiveness and metastasis **A.** Rab1A overexpression promotes HCC cell migration. Migration of SK-HEP-1 and BEL7402 cells overexpressing Rab1A or carrying a control vector was analyzed by the wound-healing assay. Left panel shows representative images taken at 0 hr (upper panels) and 48 hr (lower panels) after scratching. Right panel shows quantification of cell migration results that are expressed as the mean ± SD of three independent experiments (Student's *t*-test, *p* < 0.001). **B.** Transwell cell invasion assay was used to measure invasiveness of SK-HEP-1 and BEL7402 cells overexpressing Rab1A or carrying a control vector. Left panel shows representative images of cells that migrated through the PET membrane (magnification 200 x). Right panel shows quantification of cell invasion data. Results are expressed as mean ± SD of three independent experiments (Student's *t*-test, *p* < 0.001). **C.** Tail vein metastasis assay of SK-HEP-1 cells overexpressing Rab1A or carrying a control vector. Representative images show lungs with metastatic HCC tumors. Arrows indicate tumor nodules at the surface of the lungs. **D.** Numbers of metastatic nodules in the lungs (*p* < 0.001, independent Student's *t*-test) are shown. **E.** Representative images of H&E sections derived from metastatic lung tissue sections (magnification 100×).

Intriguingly, Rab1A expression is nearly 3 times higher in the metastatic cell line MHCC97H than the paired non-metastatic cell line MHCC97L from the same patient (Figure 1B and 1C), suggesting that Rab1A expression is related to metastasis. We hence interrogated the effect of Rab1A overexpression on HCC cell migration and invasion. Indeed, Rab1A-overexpressing SK-HEP-1 and BEL-7402 cells migrate faster and are more invasive than control cells, as determined by wound healing and transwell assays, respectively (Figure 3A and 3B). To assess the impact of Rab1A overexpression on HCC metastasis *in vivo*, SK-HEP-1 cells overexpressing Rab1A or carrying control vector were injected into the tail vein of mice. Eight weeks after injection, the animals were sacrificed, and the numbers of metastatic nodules in the lung were counted. A much greater number of metastatic nodules are found on the surface of the lung in Rab1A-overexpressing HCC cells than the control (Figure 3C and 3D). Rab1A-overexpressing tumors are also considerably larger than the control (Figure 3E). These observations indicate that Rab1A overexpression promotes HCC migration, invasion and metastasis.

### Rab1A down-regulation inhibits malignant properties of HCC

**Figure 4:**
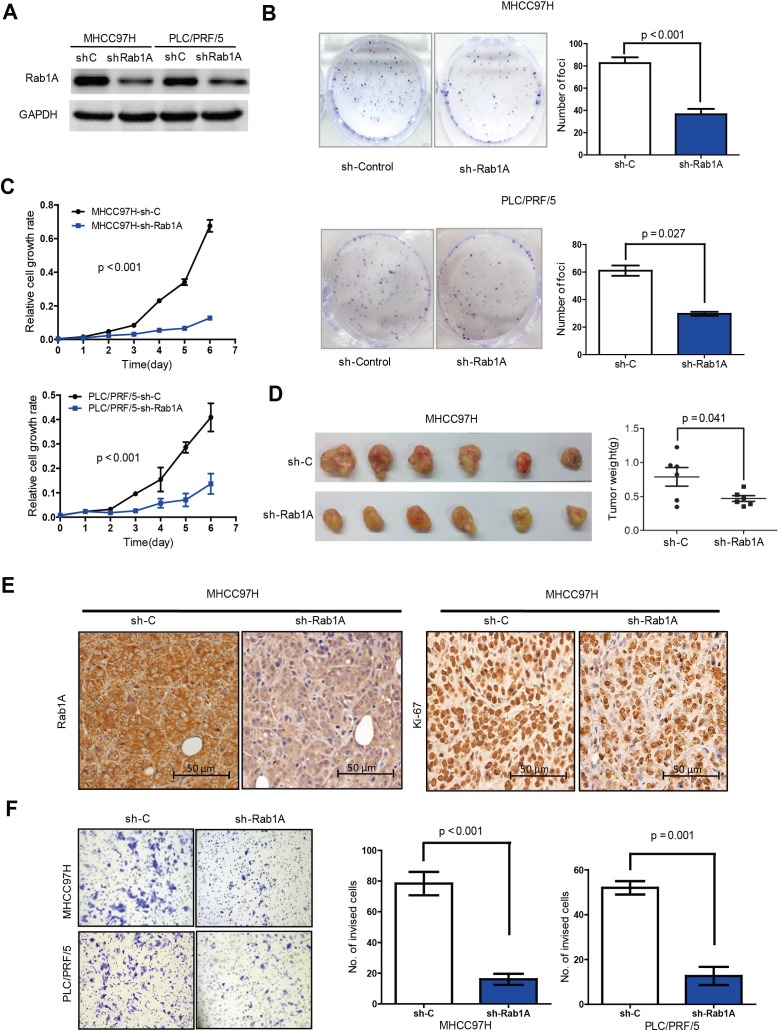
Rab1A knockdown inhibits oncogenic growth and migration of HCC cells **A.** Immunoblot analysis of Rab1A knockdown in MHCC97H and PLC/PRF/5 cells. Scrambled shRNA was used as a negative control (sh-C), and GAPDH was used as a loading control. **B.** Rab1A knockdown inhibits colony formation of MHCC97H and PLC/PRF/5 cells. Results are expressed as the mean ± SD of three independent experiments (*p* < 0.001, independent Student's *t*-test). **C.** Rab1A knockdown inhibits growth of MHCC97H and PLC/PRF/5 cells. Results are expressed as mean ± SD of three independent experiments. **D.** Rab1A knockdown attenuates HCC tumor growth in xenograft nude mice derived from MHCC97H cells. Left panels show images of xenograft tumors dissected at the end of the study. Right panels show the weights of the individual tumors. **E.** Rab1A knockdown inhibits proliferation of HCC cells in xenograft tumors derived from MHCC97-H cells expressing Rab1A shRNA or a control shRNA. Tumor sections were analyzed by IHC staining with Rab1A and Ki67 antibodies magnification 200x. **F.** Transwell cell invasion assay was used to measure cell invasion of MHCC97H and PLC/PRF/5cells expressing Rab1A shRNA or a control shRNA. Left panels show representative images of cells migrated through the PET membrane (magnification ×200). Right panels show quantification of cell invasion data. Results are expressed as mean ± SD of three independent experiments (Student's *t*-test, *p* = 0.001).

To further investigate the importance of Rab1A overexpression in HCC, we knocked down Rab1A with lentiviral shRNA in MHCC97H and PLC/PRF/5 (Figure 4A), two HCC cell lines with high Rab1A expression. Rab1A knockdown significantly inhibits their growth and colony formation (Figure 4B and 4C). We also generated xenograft tumors in nude mice with MHCC97H stably expressing a previously validated Rab1A shRNA or a scrambled control shRNA by injecting HCC cells subcutaneously into the left and right dorsal flanks of athymic nude mice. Rab1A knockdown attenuates the growth of the xenograft tumors compared with control tumors (Figure 4D). Consistently, Rab1A-knockdown tumors also display diminished proliferation as measured by lower Ki67 staining (Figure 4E). Moreover, inhibition of Rab1A expression leads to much reduced cell invasiveness (Figure 4F). These data show that Rab1A is required for malignant growth, proliferation and invasion of HCC cells.

### Rab1A promotes oncogenic growth in HCC by stimulating mTORC1 signaling

**Figure 5:**
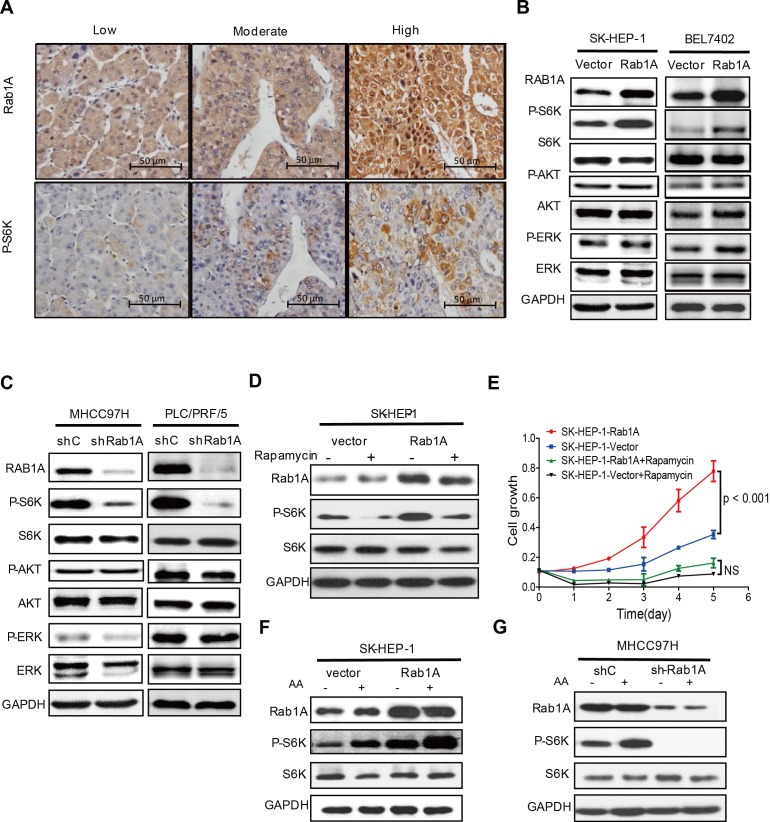
Rab1A overexpression potentiates AA-mTORC1 signaling and mTORC1-dependent oncogenic growth of HCC cells **A.** Rab1A overexpression correlates with elevated mTORC1 signaling in primary HCCs. Consecutive sections of HCCs representative of low, moderate and high Rab1A expression were analyzed by IHC to determine the level of Rab1A and P-S6K (magnification 200 ×). **B.** Rab1A overexpression stimulates mTORC1 signaling in HCC cells. Rab1A was overexpressed in SK-HEP-1 and BEL-7402cells. The effect of Rab1A overexpression on the levels of Rab1A, P-S6K, S6K, P-AKT, AKT, P-ERK and ERK was analyzed by immunoblot. GAPDH was used as a loading control. **C.** Rab1A is required for mTORC1 signaling in HCC cells. Rab1A was knocked down in MHCC97H and PLC/PRF/5cells. The effect of Rab1A knockdown on the level of Rab1A, P-S6K, S6K, P-AKT, AKT, P-ERK and ERK was analyzed by immunoblot. GAPDH was used as a loading control. **D.** Rapamycin inhibits mTORC1 signaling stimulated by Rab1A overexpression. Rab1A-overexpressing or control SK-HEP-1 cells were treated without or with 20 ng/ml of rapamycin. P-S6K was analyzed by immunoblot analysis. GAPDH was used as a loading control. **E.** Rapamycin blunts hyperactive HCC growth stimulated by Rab1A overexpression. Rab1A-overexpressing or control SK-HEP-1 cells were treated with or without 20 ng/ml of rapamycin. Cell growth was measured using the CCK-8 assay. **F.** Rab1A overexpressed enhances mTORC1 activation by AA in HCC. Rab1A was overexpressed in SK-HEP-1 cells. The effect of Rab1A overexpression on stimulation of mTORC1 by AA was determined by immunoblot of P-S6K before and after AA stimulation. GAPDH was used as a loading control. **G.** Rab1A is required for AA to stimulate mTORC1 signaling in HCC. Rab1A was knocked down in MHCC97H cells. The effect of Rab1A knockdown on stimulation of mTORC1 by AA was determined by immunoblot of P-S6K before and after AA stimulation. GAPDH was used as a loading control.

Because Rab1A is critical activator of mTORC1, we investigated the relationship between Rab1A expression and mTORC1 signaling in primary human HCC by using IHC to examine Rab1A expression in consecutive sections of the same HCC tissue. In support of a key role for Rab1A in activation of mTORC1 signaling in liver cancer, Rab1A expression in HCC tissues is generally correlated closely with the level of P-S6K (Figure 5A). To further investigate this phenomenon, we modulated Rab1A expression in HCC cell lines. Overexpression of Rab1A in SK-HEP-1 cells and BEL-7402 cells, two HCC cell lines with low endogenous Rab1A, causes a significant increase in mTORC1 signaling, as determined by phosphorylation of S6K (Figure 5B). In contrast, phosphorylation of AKT, a substrate of mTORC2 is not affected. Additionally, phosphorylation of ERK, another major mitogenic kinase, remains unchanged. Conversely, Rab1A knockdown in MHCC97H and PLC/PRF/5 cells, two HCC cell lines with high endogenous Rab1A, attenuates mTORC1 signaling, but not mTORC2 or ERK signaling (Figure 5C). These results indicate that Rab1A is an activator of mTORC1 signaling in HCC. To determine whether the hyper-activation of mTORC1 signaling is important for the oncogenic activity of Rab1A, Rab1A-overexpressing SK-HEP-1 cells were treated with rapamycin. Rapamycin blunts Rab1A-stimulated mTORC1 signaling and HCC cell growth (Figure 5D and 5E). This result indicates that activation of mTORC1 signaling is critically important for Rab1A to promote the oncogenic growth of HCC and that Rab1A-stimulated growth is sensitive to the anticancer drug rapamycin.

### Rab1A hyper-activates AA-mTORC1 signaling in HCC

mTORC1 is an evolutionarily conserved nutrient sensor whose activity is regulated by AA sufficiency. To ask if Rab1A regulates mTORC1 signaling in HCC, we examined the dependency of mTORC1 activation on Rab1A by AA. We found that overexpression of Rab1A strongly potentiate the ability of AA to activate mTORC1 as shown by much enhanced S6K1 phosphorylation in SK-HEP-1 cells (Figure 5F). On the other hand, Rab1A knockdown blunts the ability of AA to stimulate phosphorylation of S6K1 in MHCC97H cells (Figure 5G). These observations demonstrate that Rab1A is essential for mTORC1 activation by AA and that Rab1A overexpression leads to hyperactive AA-mTORC1 signaling in HCC. Taken together, our data suggest that increased Rab1A expression promotes tumorigenesis and metastasis in HCC through activation of AA-dependent mTOR signal transduction.

### Rab1A is overexpressed in human primary HCCs

**Figure 6:**
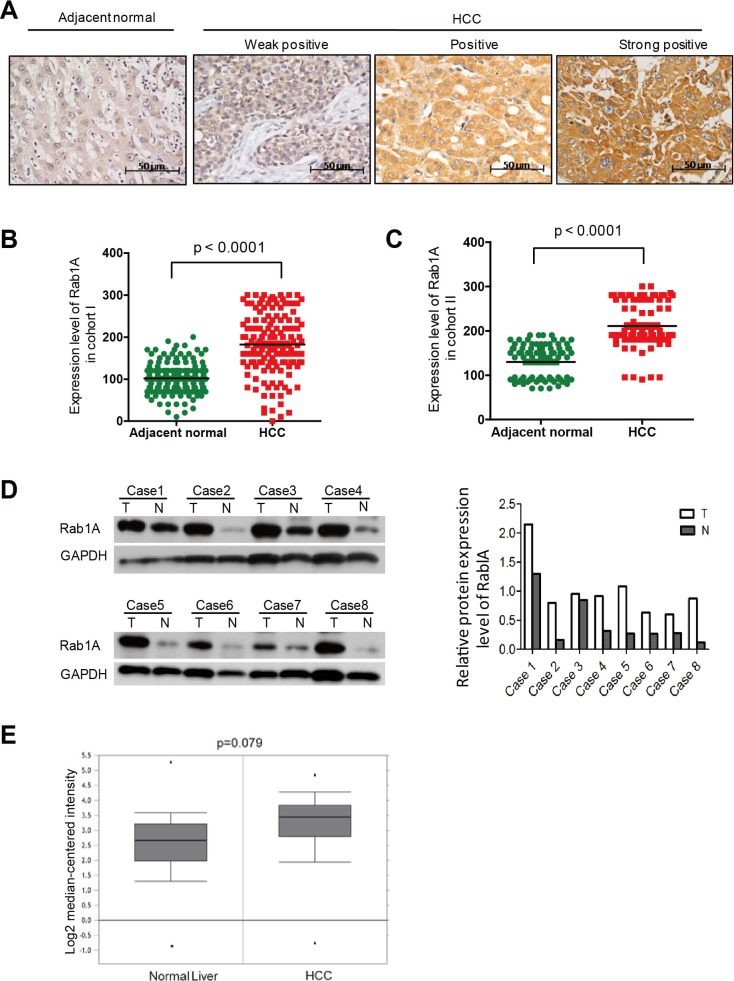
Rab1A is overexpressed in HCC, which is associated with a poor prognosis **A.** Representative immunohistochemistry (IHC) staining images of primary HCC tissues with weak, moderate and high positivity for Rab1A expression, and a representative non-cancerous liver tissue (original magnification, 200×). **B.** Scatter plots of IHC scores for Rab1A protein expression in HCC and non-cancerous liver tissues in Cohort I. **C.** Scatter plots of IHC scores for Rab1A protein expression in HCC and non-cancerous liver tissues in Cohort II. **D.** Immunoblot analysis of Rab1A protein expression in eight pairs of randomly selected HCC and matching non-cancerous liver tissue samples. GAPDH is used as a loading control. **E.** Rab1A mRNA expression is increased in HCC. Shown is the expression of Rab1A mRNA as determined by analysis of the genomic gene expression profile in 225 HCC and 220 normal liver tissue samples by Roesseler et al. Data is downloaded from OncoMine (www.oncomine.com).

To investigate the clinical significance of Rab1A expression in HCC, we analyzed its expression by immunohistochemistry (IHC) in 143 paired primary HCC and adjacent non-cancerous liver tissues from South China (Guangzhou) (Cohort I). The data shows that Rab1A expression is significantly up-regulated in tumors compared with adjacent non-cancerous tissues (Figure 6A and 6B). To verify the result in a second, independent cohort, we further examined 90 paired primary HCC and adjacent non-cancerous liver tissues from East China (Shanghai) (cohort II). Essentially the same result is seen with Cohort II (Figure 6C). We further performed an immunoblot analysis of 8 randomly selected pairs of HCC and matching adjacent normal liver tissues. Rab1A protein expression in HCC is higher in seven out of eight cases after adjusting against the GAPDH loading control, confirming the IHC staining results (Figure 6D). In addition, we found that Rab1A mRNA is also increased in 225 HCC samples versus 220 normal liver tissues by analysis of an Oncomine mRNA expression profile, which represents yet another independent source of samples (Figure 6E). Together, these findings demonstrate that Rab1A is frequently overexpressed in HCC.

### Rab1A overexpression is significantly associated with poor prognosis in HCC

**Figure 7:**
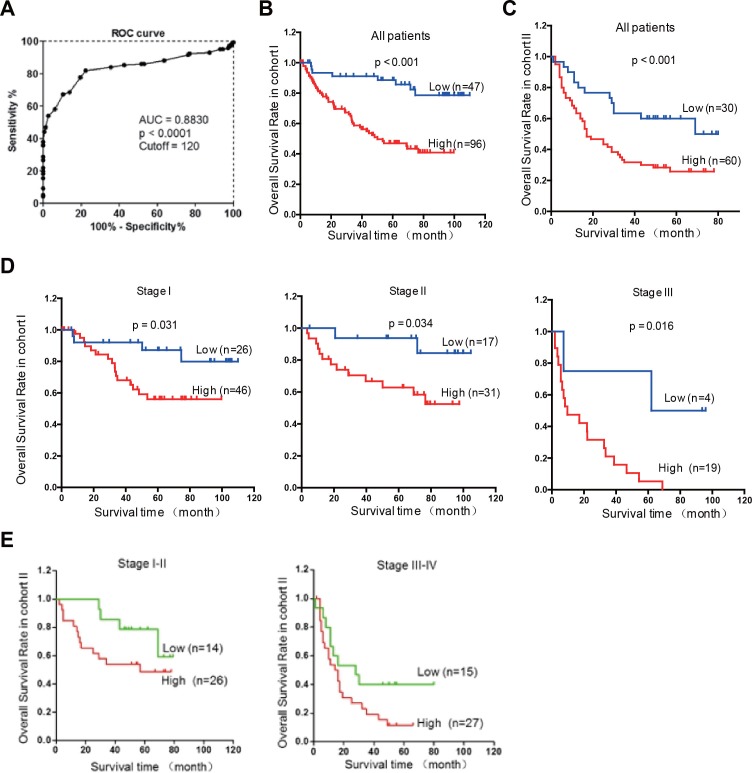
Rab1A overexpression is associated with a poor prognosis of HCC **A.** ROC curve was used to determine the best cutoff expression score of Rab1A for distinguishing HCC from non-tumor controls. AUC, Area Under roc Curve. **B.** Kaplan-Meier survival analysis comparing the overall survival time of HCC patients with low or high Rab1A protein expression in Cohort I. **C.** Kaplan-Meier survival analysis comparing the overall survival time of HCC patients with low or high Rab1A protein expression in Cohort II. **D.** Kaplan-Meier survival analysis comparing the overall survival time of patients at different HCC stages with low or high Rab1A protein expression in Cohort I. **E.** Kaplan-Meier survival analysis comparing the overall survival time of patients at different HCC stages with low or high Rab1A protein expression in Cohort II.

To evaluate the clinical significance of Rab1A overexpression, we analyzed the relationship between Rab1A IHC scores and clinicopathological features of HCC patients in cohorts I and II. According to the ROC curve, we defined IHC score 120 as cutoff value to distinguish Rab1A expression level (Figure 7A). There is no statistically significant correlation between Rab1A high expression and clinicopathological features such as age, gender, tumor diameter, pathological stage, hepatitis B virus infection, recurrence, TNM stage (Table 1). Kaplan-Meier analysis reveals that overall survival of HCC patients with high Rab1A expression is significantly worse than those with low Rab1A expression in cohorts I and II (Figure 7B and 7C). Furthermore, high Rab1A expression correlates with poor prognosis at all three TNM stages (Figure 7D) in cohort I. Thus, Rab1A level can provide predictive value for the outcome of HCC patients. The relationship between Rab1A expression and overall survival of HCC patents was validated in cohort II (Figure 7E). Univariate Cox regression analysis further supports the finding that Rab1A overexpression is significantly associated with an elevated risk of HCC-related death (Table 1). The relative risk (RR) of high Rab1A expression was 7.863, with a 95% confidence interval (CI) ranging from 3.128-19.768 (*p* < 0.001) in cohort I. To adjust for potential confounding factors, multivariate Cox regression was performed on all variables (Table 1). The results show that Rab1A expression is an independent prognostic factor for HCC patients (Table 1). Analysis of the results in cohort II leads to the same conclusion. These results robustly demonstrate that Rab1A is an independent prognostic predictor of poor survival in patients with HCC. As expected, the TNM stage and recurrence are also independent prognostic indicators for HCC patient (Table 2).

**Table 1:** Relationship between Rab1A expression and clinicopathologic features in cohort I and cohort II

Clinical parameters	Rab1A Level in Cohort I			Rab1A Level in Cohort II		
	Low n (%)	High n (%)	p value	Low n (%)	High n (%)	p value
Age (years)						
–≤60	45 (69.2)	53 (67.9)	0.883	30 (73.2)	37 (75.5)	0.795
–＞60	20 (30.8)	25 (32.1)		11 (26.8)	12 (24.5)	
Gender						
–Male	40 (85.1)	81 (84.4)	0.909	29 (96.7)	52 (86.7)	0.136
–Female	7 (14.9)	15 (15.6)		1 (3.3)	8 (13.3)	
TNM stage						
–I & II	43 (91.5)	77 (80.2)	0.085	15 (50.0)	33 (55.0)	0.349
–III	4 (8.5)	19 (19.8)		15(50.0)	27 (45.0)	
Pathological Grade						
–I&II	38 (80.9)	66 (68.8)	0.127	21 (70.0)	36 (60.0)	0.353
–III&IV	9 (19.1)	30 (31.2)		9 (30.0)	24 (40.0)	
Tumor Number						
–Multiple (＞1)	40 (85.1)	68 (70.8)	0.062	29 (96.7)	53 (88.3)	0.19
–Single (1)	7 (14.9)	28 (29.2)		1 (3.3)	7 (11.7)	
Tumor Size (cm)						
–Large (＞5)	32 (68.1)	65 (67.7)	0.964	16 (53.3)	37 (61.7)	0.449
–Small (≤5)	15 (31.9)	31 (32.3)		14 (46.7)	23 (38.3)	
Liver cirrhosis						
–Yes	33 (71.7)	67 (69.1)	0.921			
–No	13 (28.3)	30 (30.9)				
Serum AFP (μg/L)						
–≤20	23 (48.9)	21 (21.9)	<0.001			
–＞20	24 (51.1)	75 (78.1)				
HbsAg						
–Positive	25 (53.2)	90 (93.8)	0.614			
–Negative	22 (46.8)	6 (6.2)				
CEA (ng/ml)						
–＞5	29 (61.7)	86 (89.6)	0.698			
–≤5	18 (38.3)	10 (10.4)				
Recurrence						
–Yes	8 (17.0)	21 (21.9)	0.498			
–No	39 (83.0)	75 (78.1)				

**Table 2:** Cox regression analysis of Rab1A expression and clinicopathologic features in cohort I and cohort II

Clinical parameters	Univariate analysis			Multivariate analysis		
	RR	95% CI	p value	RR	95% CI	p value
**Chort I**						
Rab1A Exp (High vs Low)	7.863	3.128-19.768	<0.001	7.247	2.833-18.539	<0.001
Age (≤60 vs >60)	0.982	0.961-1.004	0.114	0.999	0.978-1.020	0.911
Gender (Female vs Male)	0.810	0.409-1.607	0.547	1.065	0.490-2.312	0.874
TNM Stage (III vs I-II)	5.613	3.242-9.719	<0.001	4.104	2.041-8.251	<0.001
Tumor Size (cm) (>5 vs ≤5)	1.684	0.931-3.044	0.085	1.210	0.635-2.306	0.561
Tumor Number (>1 vs 1)	3.099	1.803-5.325	<0.001	1.069	0.727-1.574	0.734
Recurrence (Yes vs No)	2.895	1.657-5.057	<0.001	2.612	1.450-4.705	0.002
**Chort II**						
Rab1A Exp (High vs Low)	1.726	1.465-2.986	0.009	2.289	1.224-4.281	0.010
Age (≤60 vs >60)	0.929	0.551-1.566	0.782	0.962	0.570-1.625	0.885
Gender (Female vs Male)	1.253	0.500-3.141	0.619	1.327	0.522-3.370	0.353
TNM Stage (III vs I-II)	2.409	1.410-4.116	0.001	2.342	1.260-4.351	0.007
tumor Size (cm) (>5 vs ≤5)	2.106	1.195-3.709	0.010	1.212	0.633-2.323	0.562
Tumor Number (>1 vs 1)	1.514	0.648-3.533	0.338			

## DISCUSSION

Rab1A is a small GTPase well known for its role in regulating ER-to-Golgi vesicular transport [24]. Although it is generally thought to be a housekeeping enzyme, recent evidence indicates that Rab1A is closely linked to human diseases. For example, Rab1A is critical for infection by the pathogen *Legionella pneumophila* [25, 26]. A DNA microarray study in a murine dilated cardiomyopathy model identified Rab1A as one of the highly induced genes during disease progression [27]. In the same study, Rab1A transgene is shown to cause cardiac hypertrophy in a gene dosage-dependent manner [27]. Another microarray expression profiling of human tongue squamous carcinomas finds that Rab1A is highly expressed in tongue cancer [28], although the significance of Rab1A overexpression in this particular malignancy remains unknown. The last two studies indicate that aberrant Rab1A expression is closely associated with hyper-proliferative diseases. However, it remains unclear whether Rab1A is overexpressed in common human cancers, and if so, what the significance and mechanism of Rab1A overexpression are in the pathobiology of cancer. Rab1A is recently recognized as an alternative branch of AA signaling to activate mTORC1 independently of Rag GTPases [29, 30]. It is anchored in the ER and Golgi apparatus, instead of lysosomes for Rag GTPases, which is consistent with that mTOR is found in these organelles in many different types of cancer cells [31, 32]. Our results demonstrate that aberrant AA signaling is a cancer-driving event for common malignancies such as colorectal and liver cancers.

This study shows that Rab1A is frequently overexpressed in human primary HCCs. Significantly, HCC cases with high Rab1A expression suffer poor prognosis. A Cox regression analysis indicates that Rab1A protein level is a strong independent prognostic biomarker for HCC patients. Higher Rab1A expression in HCC is correlated closely with the increased Rab1A DNA copy number. In contrast, no significant Rab1A promoter methylation is associated with HCC, indicating that Rab1A overexpression in HCC primarily results from gene amplification. When overexpressed, Rab1A promotes oncogenic growth, invasion and metastasis of HCC cells. Conversely, down-regulation of Rab1A attenuates these malignant phenotypes of HCC. Thus Rab1A acts as an oncogene to promote malignant liver cell growth and metastasis. These observations further suggest that activation of AA signaling can promote both cancer growth and metastasis.

mTORC1 is a master regulator of cell growth and metabolism in response to nutrient signals, particularly AAs. AAs are essential nutrients that serve as precursors for protein synthesis and for other catabolism. In recent years, it is increasingly recognized that AAs are also important mitogenic signals stimulating cellular growth and metabolism. Rag small GTPases have previously been shown to be mediators of AA signaling [33, 34]. However, we found that AAs are still capable of activating mTORC1 in the absence of Rag GTPases in yeast and human embryonic kidney cells, and this Rag-independent mechanism is dependent on Rab1A [21]. Here we show that Rab1A is also crucial for AA to stimulate stimulate mTORC1 signaling in HCC. Importantly, Rab1A overexpression enhances AA-mTORC1 signaling in hepatocellular carcinomas, suggesting that aberrant AA signaling is a main driving event for hepato-oncogenesis. Because Rab1A overexpressing HCC cells are sensitive to rapamycin, Rab1A can be potentially used as a surrogate biomarker to guide personalized, mTORC1-targeted HCC therapy. Further study of Rab1A should have considerable basic and translational significance.

## EXPERIMENTAL PROCEDURES

### Patients and HCC samples

Two cohorts of archived de-identified specimens were used in this research: Cohort I of 143 HCC patients who did not receive preoperative radiotherapy or chemotherapy was selected consecutively from the surgical pathology archives of Sun Yat-Sen University Cancer Center (Guangzhou, China) with a median follow-up of 48 months (Ranging from 4 to 111 months). These patients underwent radical hepatectomy between 2005 and 2008. Cohort II of 90 HCC patients was obtained with the same selection criteria from Zhong Shan Hospital (Shanghai, China). These patients received radical hepatectomy between 2007 and 2009, with a median follow-up of 33 months (range: 2-80 months). All tumor samples were analyzed by two experienced pathologists. The overall survival time was calculated from the date of surgery to the date of death due to any cause or last follow-up. This study was approved by the Committees for Ethical Review of Research at Sun Yat-Sen University and the Zhong Shan Hospital research ethics committee. Informed consents signed by the patients were collected.

### Cell lines and cell culture

Liver cancer cell lines QGY-7703, PLC/PRF/5, MHCC-97H, MHCC-97L, and immortalized hepatocyte QSG-7701 were maintained in DMEM (Gibco BRL, Grand Island, New York, USA) plus 10% fetal bovine serum (Gibco BRL). Liver cancer cell lines BEL-7402 and SK-HEP-1, and immortalized hepatic epithelial cell line LO2 were cultured in RPMI1640 with 10% fetal bovine serum.

### RNA extraction and qRT-PCR

TRIZOL Reagent (Invitrogen) was used to isolate total RNA. To quantify mRNA expression level in cells, the Advantage RT-for-PCR kit (Clontech) was used to synthesize cDNA. The SYBR Green PCR master mix (Applied Biosystems) was then used for qPCR, which was followed by detection with an ABI PRISM 7900 Sequence Detector and analysis with the ABI SDS 2.3 software (Applied Biosystems). Relative Rab1A expression level (defined as fold change) was expressed as 2^−ΔCT^ (ΔC_T_ = C_T_^Rab1A^ − C_T_^GAPDH^) and normalized to the relative expression level detected in control cells. Each sample was tested in triplicate. Primer sequences are listed in Supplementary Table 1.

### Methylation-specific PCR (MSP)

For MSP assay, genomic DNA was extracted from HCC cells with the DNeasy Tissue Kit (Qiagen) according to manufacturer's instructions. Genomic DNA underwent bisulfite modification with the EZ DNA Methylation-Direct Kit (Zymo Research, Orange, CA). Primer sequences for bisulfite genomic sequencing (BGS) and MSP were listed in Supplementary Table 1. BGS PCR fragments were analyzed by Sanger sequencing method (Invitrogen).

### Plasmids and DNA transfection

Human Rab1A cDNA plasmids were described previously [21]. Rab1A plasmid was transfected into SK-HEP-1 and BEL7402 cells using Lipofectamine 2000 (Invitrogen) according to manufacturer's instructions. Stable Rab1A-expressing or control cell clones were selected in culture medium containing G418 (Roche Diagnostics) at a concentration of 300 μg/ml. Lentiviral containing short hairpin RNAs (shRNA) targeting Rab1A was purchased from Thermo Fisher and were used to infect MHCC-97H and PLC/PRF/5 cells according to manufacturer's instructions. Cells were also infected with scrambled control shRNA as controls. Puromycin was used to select stable clones.

### Wound healing and transwell invasion assays

Cell mobility was measured by the scratch wound-healing assay. Briefly, HCC cells expressing Rab1A or carrying the control vector were cultured in six-well plates until confluence, and then scratched with a 10 μl pipette tip. Cell migration images were captured at 0 hr and 48 hr after scratching. Each sample was analyzed in triplicate. Cell invasion assay was performed with BD BioCoat Matrigel Invasion Chambers (Becton Dickinson Labware) following manufacturer's instruction. Cells were seeded at proper number. The chambers were incubated for 16-18 h at 37°C in 5% CO_2_ atmosphere. The upper surface of the membrane was scrubbed carefully with a cotton swab to remove the remaining cells and matrigel matrix. Cells on the lower surface of the scrubbed membranes were fixed with 100% methanol and stained with 0.5% crystal violet. Five random fields were counted under the light microscope.

### Analysis of Rab1A copy number

The cancer genomic databases Oncomine (https://www.oncomine.org/) and The Cancer Genome Atlas (TCGA,http://tcga-data.nci.nih.gov/tcga/) were used to analyze Rab1A DNA copy number alteration and mRNA expression in HCC. The website cBioPortal (http://www.cbioportal.org/public-portal/index.do) was used to download and analyze HCC data from TCGA.

### Cell viability assay

The cells were seeded at 1×10^3^ cells per well in 96-well plates for the cell growth assay using the CCK-8 assay kit (Dojindo, Shanghai, China). All of the results are expressed as the mean ± SD.

### Focus formation assay

Cells were plated in triplicate in 6-well plates to form colonies for up to 2-4 weeks. The medium containing selective antibiotics was replaced every 3-5 days. The colonies were then stained with crystal violet and counted. The results are expressed as mean ± SD.

### Immunohistochemical staining (IHC)

The standard streptavidin-biotin-peroxidase complex method was used for IHC staining. Briefly, after tumor sections were deparaffinized, nonspecific binding was blocked with 10% normal goat serum for 10 min and endogenous peroxidase activity was blocked with 3% hydrogen peroxide (H_2_O_2_) for 10 min. For antigen retrieval, the slides were soaked in 10 mM citrate buffer (pH 6.0) and boiled for 15 min in a microwave oven. Tumor sections were then incubated in a 1:200 dilution of Rab1A-specific antibody (Santa Cruz Biotechnology, Santa Cruz, CA, USA) at 4°C overnight in a humidified chamber. After washing, tumor sections were incubated with horseradish peroxidase-conjugated anti-goat antibody (DakoCytomation, Carpentaria, CA) for 30 min at room temperature. 3,5-diaminobenzidine (DAB) substrate was used for color development, followed by Mayer's hematoxylin counterstaining. For IHC score, the percentage (0-100%) of stained tumor cells was multiplied by the intensity (0, 1, 2, or 3) to achieve a score between 0 and 300.

### Antibodies and immunoblot

Immunoblot was performed according to previously described [35, 36]. Briefly, Protein lysates were resolved on a SDS-PAGE gel, transferred onto a polyvinylidenedifluoride (PVDF) membrane (Millipore, Billerica, MA), incubated with a primary antibody, and then a secondary antibody. The signals were detected using the enhanced chemiluminescence method (GE Healthcare). Antibodies against GAPDH, P-S6K(T398), S6K, P-AKT(S473), AKT, P-ERK(T202, Y204), andERK were purchased from Cell Signaling Technology, and antibodies anti-Rab1A and anti-P-S6K(T398) for Immunohistochemistry were purchased from Santa Cruz and Abcam, respectively.

### AA starvation or stimulation assay

Amino acid starvation and stimulation assay were performed according to the previous study [21].

### Metastasis assay

All animal procedures were performed in accordance with the Guide for the Care and Use of Laboratory Animals (NIH publications Nos. 80-23, revised 1996) and the Institutional Ethical Guidelines for Animal Experiments developed by Sun Yat-sen University. Four groups of 6 mice were each given intravenous tail vein injection with 2×10^6^ cells. After 8 weeks of observation, mice were sacrificed and tumor nodules on the lung surfaces were counted, excised and embedded in paraffin.

### Statistical analysis

Data are expressed as means ± SEM. SPSS 13.0 was used for all data analyses. Clinical correlations were analyzed by Pearson's chi-square test and Fisher's exact probabilities. Receiver operating characteristic (ROC) curve was used to estimate the cutoff value of Rab1A expression. Survival analyses were performed using Kaplan-Meier plots and the log-rank test. Univariate and multivariate survival analyses were conducted using a Cox proportional hazards regression model. Differences are considered significant when *p* value is less than 0.05.
